# Xanthohumol exerts anti-inflammatory effects in an in vitro model of mechanically stimulated cementoblasts

**DOI:** 10.1038/s41598-022-19220-6

**Published:** 2022-09-02

**Authors:** Christian Niederau, Shruti Bhargava, Rebekka Schneider-Kramman, Joachim Jankowski, Rogerio B. Craveiro, Michael Wolf

**Affiliations:** 1 Department of Orthodontics, Dental Clinic, University Hospital RWTH, Pauwelsstr. 30, 52074 Aachen, Germany; 2 Institute for Cell Biology, University Hospital RWTH, Pauwelsstr. 30, 52074 Aachen, Germany; 3 Institute for Molecular Cardiovascular Research, University Hospital RWTH, Pauwelsstr. 30, 52074 Aachen, Germany

**Keywords:** Cell biology, Cell signalling, Cytokines, Kinases

## Abstract

Xanthohumol (XN) is a prenylated plant polyphenol that naturally occurs in hops and its products, e.g. beer. It has shown to have anti-inflammatory and angiogenesis inhibiting effects and it prevents the proliferation of cancer cells. These effects could be in particular interesting for processes within the periodontal ligament, as previous studies have shown that orthodontic tooth movement is associated with a sterile inflammatory reaction. Based on this, the study evaluates the anti-inflammatory effect of XN in cementoblasts in an in vitro model of the early phase of orthodontic tooth movement by compressive stimulation. XN shows a concentration-dependent influence on cell viability. Low concentrations between 0.2 and 0.8 µM increase viability, while high concentrations between 4 and 8 µM cause a significant decrease in viability. Compressive force induces an upregulation of pro-inflammatory gene (*Il-6, Cox2, Vegfa*) and protein (IL-6) expression. XN significantly reduces compression related IL-6 protein and gene expression. Furthermore, the expression of phosphorylated ERK and AKT under compression was upregulated while XN re-established the expression to a level similar to control. Accordingly, we demonstrated a selective anti-inflammatory effect of XN in cementoblasts. Our findings provide the base for further examination of XN in modulation of inflammation during orthodontic therapy and treatment of periodontitis.

## Introduction

Xanthohumol (XN), a prenylated chalcone is found in hops (*Humulus lupulus* L.) and in its’ major product beer. After the first discovery in 1913 and revelation of the chemical structure in 1961, many years elapsed until XN moved into the focus of science at the beginning of twenty-first century. Many reports about diverse capabilities have been published in the past 20 years. Especially in cancer research, XN exhibits interesting cytotoxic and anti-angiogenic properties but also anti-inflammatory effects^1,2^. Nevertheless, the exact molecular target of XN remains still unknown.

In orthodontic therapy, teeth are moved through the alveolar bone to enhance functional and aesthetic properties of the stomato-gnathic system. For this purpose, therapeutic forces are applied to teeth, resulting in an initial deflection of a tooth inside its alveolar socket^3^. As a result, the periodontal ligament (PDL), a structure between tooth root and alveolar bone, can be divided into areas of compression and tension. Compressed areas react with bone resorption whereas tensed areas show bone apposition, which enables the tooth to move along the vector of applied force. This PDL represents a microenvironment, consisting of multiple different cell types. At the alveolar bone side, osteoblasts and osteoclasts permit bone remodeling. Collagen-like Sharpeys’ Fibres produced by fibroblasts (PDLF) connect alveolar bone and cementum. Cementum, produced by cementoblasts, represents the essential structure for attaching Sharpeys’ Fibres at the tooth root and thus for tooth anchorage^4,5^.

The mechanisms inside the PDL following mechanical stimulation have been shown in multiple in vitro and in vivo experiments. Essential for orthodontic tooth movement is a sterile inflammatory process inside the PDL. Attenuation of the inflammatory process resulted in reduced orthodontic tooth movement (OTM)^6–9^ whereas pro-inflammatory medication enhanced OTM^10^. The upregulation of several pro-inflammatory markers like interleukins (IL-6, IL-8, IL-1), prostaglandin-endoperoxide synthase 2 (PTGS2), cyclooxygenase-2 (COX-2), alkaline phosphatase (ALPL), matrix metalloproteinases (MMP) and vascular endothelial growth factor A (VEGFA) has been observed in studies with human PDLF and murine cementoblasts^11–15^. IL-6 seems to play an important role to trigger this reaction in cementoblasts. It is involved in the STAT3 pathway of RANKL induction^16^, that was shown to be crucial for osteoclast differentiation and activation^17^ which finally enables remodeling processes of the alveolar bone^18^. Nevertheless, although these regulatory effects have been investigated in detail in human PDLF, we do not know much about the reaction of these effects in cementoblasts. These cells, located on the root surface, are exposed to the same conditions in their direct proximity of PDL cells during OTM. Therefore, the capacity of cementoblasts to trigger periodontal remodeling needs to be better examined.

XN and its anti-inflammatory capacities become interesting for the modulation of the sterile inflammatory processes inside the PDL. A better understanding of the regulation of these mechanisms is particularly important for handling the known orthodontic side effects such as severe root resorptions in compressed PDL areas^19,20^ which may be related to a dysregulation inside the PDL/cementum interface. Therefore, this modulation of XN has not only a potential benefit in orthodontic therapy, but also for pathogenesis of periodontal tissues, e.g. gingivitis or periodontitis.

This work clearly demonstrates that Xanthohumol increases the sterile cellular inflammation and modulates molecular pathways caused by compressive force in cementoblasts in an in vitro model simulating the early phase of OTM. We present the first evidence that XN reduces the inflammatory reaction on mRNA and protein level. In addition, we could confirm the modulation of IL-6 expression and identify certain key cell signaling molecules involved in the production of pro-inflammatory cytokines.

Based on favorable anti-inflammatory effect of XN, we recommend further in vivo assessment in order to target the modulation of orthodontic and periodontitis therapy.

## Results

### Xanthohumol enhances and reduces the viability of cementoblasts in a time- and dose-dependent manner

**Figure 1:**
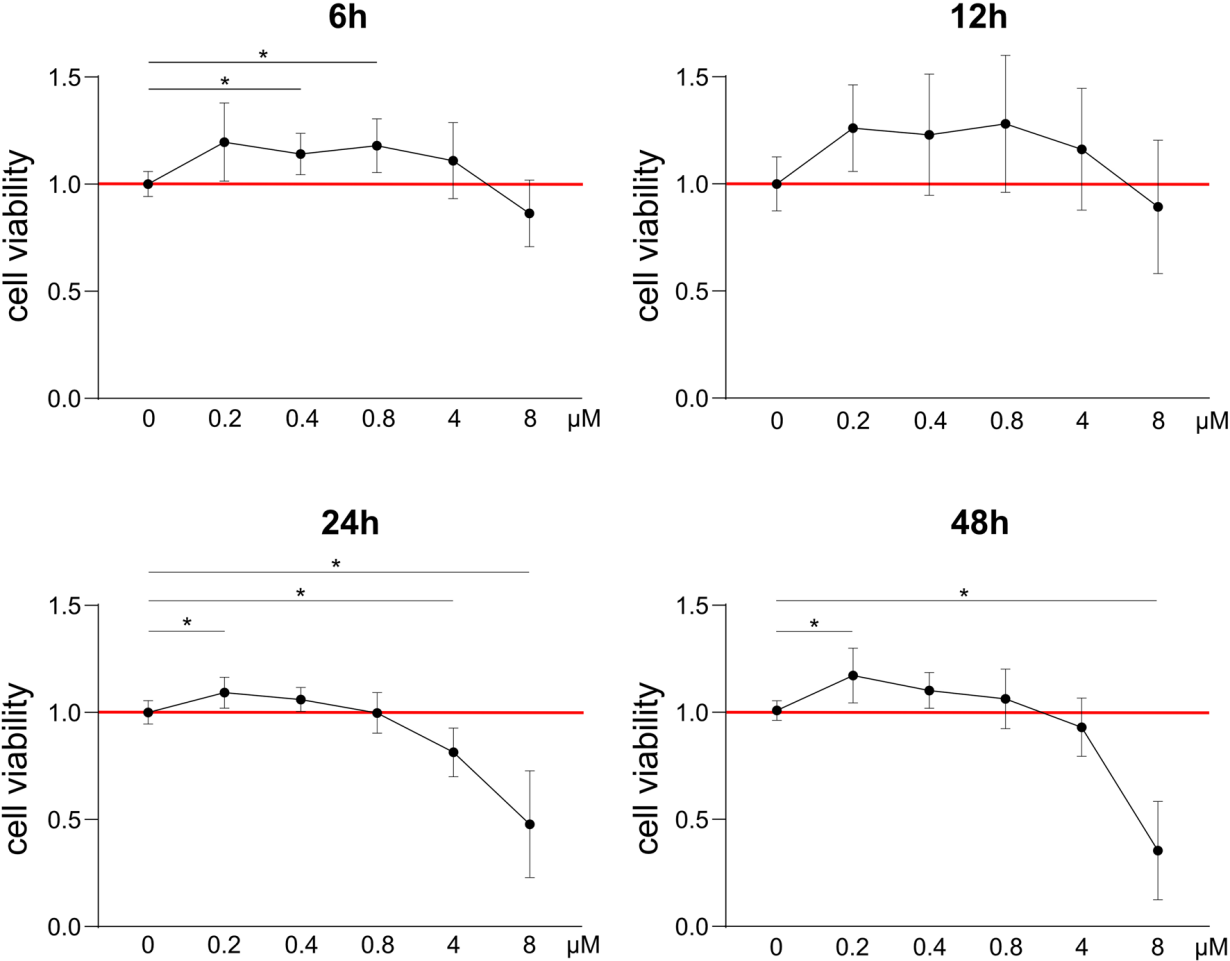
MTS-Assay with xanthohumol concentrations from 0 up to 8 µM, analyzed 6, 12, 24 and 48 h after application. Low doses enhanced cementoblast cell viability while 4 and 8 µM exerted cytotoxic effects, especially at late time points. Normalized to control (red line); *p < 0.05 was considered statistically significant by Welch-ANOVA.

In a dose response study, the cytotoxic potency of XN was assessed in cementoblast cells. For comparative assessment of cell viability by MTS assay, XN concentrations were chosen to reflect the range of different plasma levels observed in test persons for XN, namely 0.4 µM^21^. At lowest concentrations of 0.2 and 0.4 µM enhanced OCCM cell viability independent of tested time periods. At 0.8 µM the positive effect observed at 6 h (18 ± 12.5%, *p* = 0.0395) and 12 h (15 ± 17%) was no more detectable after 24 h and 48 h (6 ± 13.9%). Even 4 µM showed improved cell viability on trend, but after 24 h metabolism was decreasing (− 18.6 ± 11.3%, *p* = 0.0020). Addition of 8 µM XN always resulted in reduced cell viability with a clearly stronger effect at 24 h (− 52 ± 24.8%, *p* = 0.0003) and 48 h (− 64.3 ± 22.9%, *p* = 0.0004) compared to earlier measurements. Taken together, viability promoting effects of XN appeared already at short times whereas viability-inhibiting effects were increased at longer time points (Fig. 1).

### XN shows cell proliferation supporting effects

**Figure 2:**
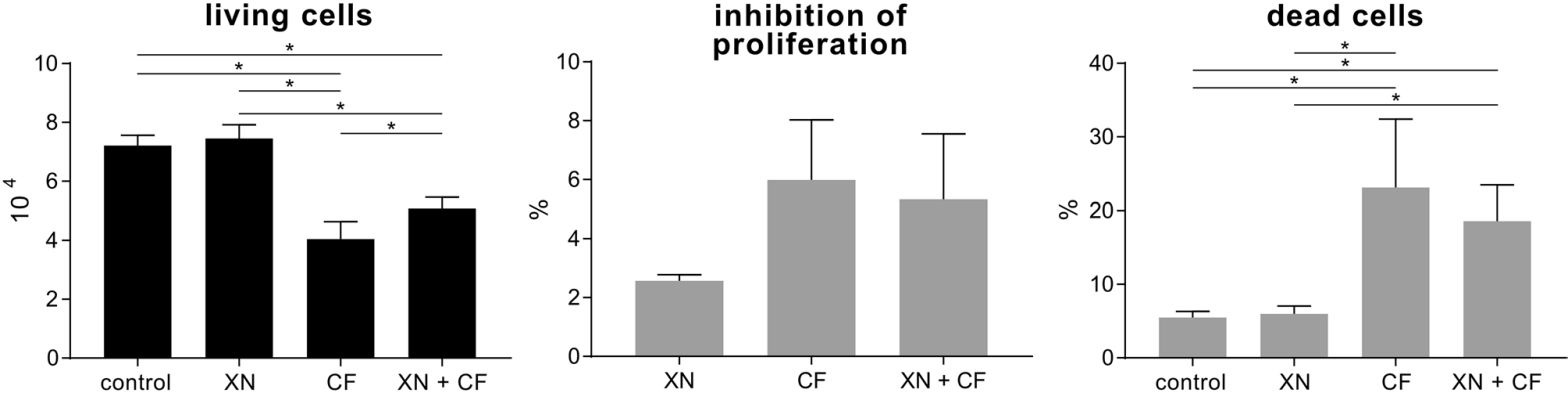
Effects of xanthohumol (XN) on proliferation and survival of mechanically stimulated OC/CM cells. OC/CM were pretreated with XN at final concentration of 0.4 µM for 24 h, followed by static compressive stimulation for 24 h. Cell count, inhibition of proliferation and dead cells were identified by flow cytometry. CF = stimulation with static compressive force; *p < 0.05 was considered statistically significant by ANOVA followed by Tukey's multiple comparisons test.

At concentrations corresponding to plasma levels, we further dissected the kinetics of the proliferative effects of XN treatment in a period of 24 h by means of a flow cytometry-based proliferation/cell death assay. Here we investigated cell proliferation and death rate of compressively stimulated cementoblasts treated with and without XN compared to control. Static compression led to a significantly lower cell count after 24 h (40,481 ± 5928, *p* < 0.0001) compared to control (72,098 ± 3548). Addition of XN did not affect total events in unstimulated conditions (74,531 ± 4676). In contrast, XN was able to inhibit the negative effect of mechanical stimulation significantly (50,890 ± 3758, *p* = 0.0324). Furthermore, compressive force resulted in increased inhibition of proliferation and dead cells number. The tendency to enhance OC/CM cell viability of doses of 0.8 µM by MTS assay was observed. An addition of XN had slightly positive but not significant effects regarding dead cells (CF 23.1 ± 9.3%, CF + XN 18.6 ± 4.9%) (Fig. 2).

### XN reduces Il-6 mRNA expression in compressively stimulated cementoblasts

**Figure 3:**
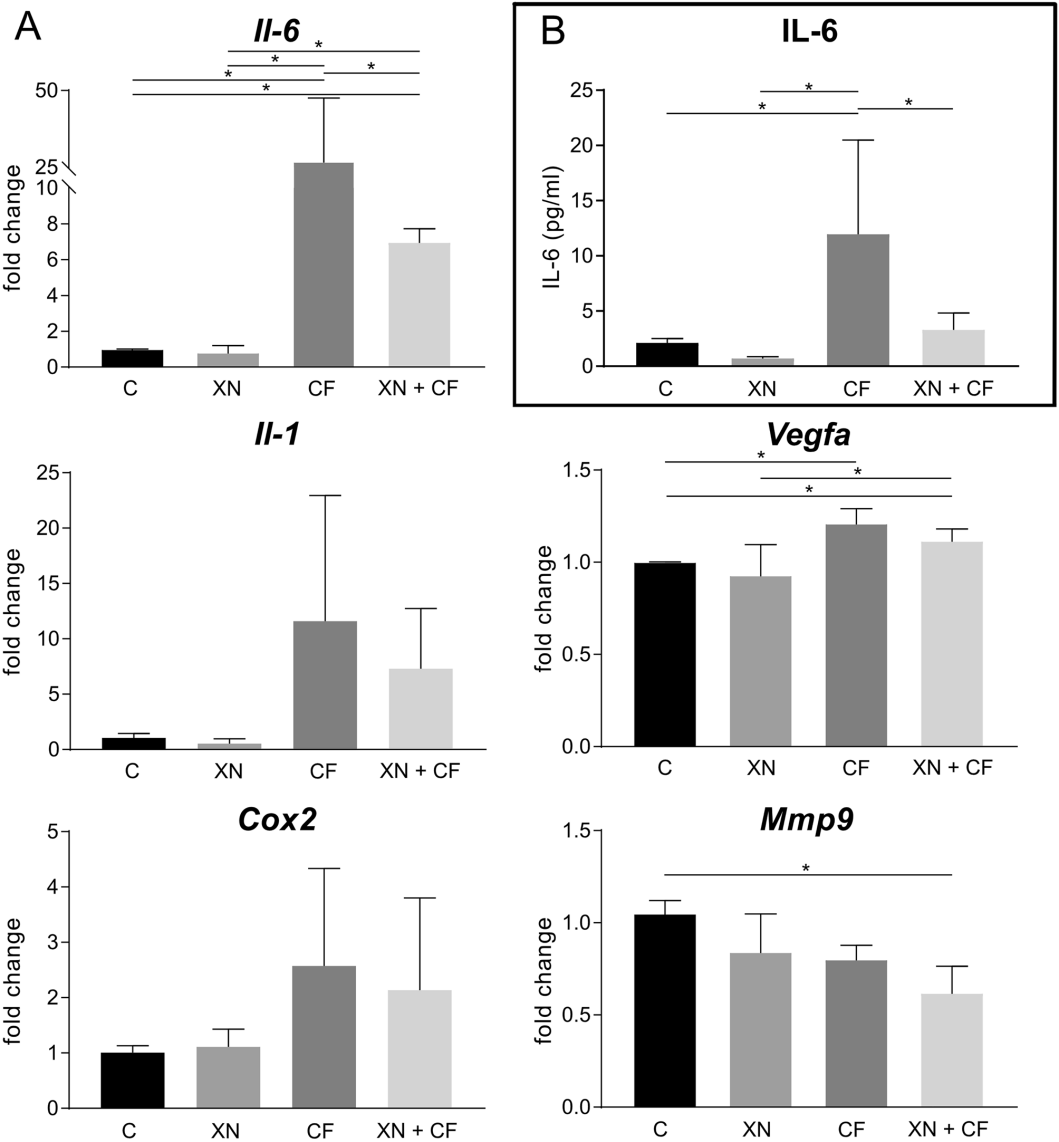
Effect of xanthohumol (XN) on OC/CM stimulated with static compressive force (CF). (**A**) Gene expression results of cytokines. Data were normalized to the reference gene *Rpl22*, shown as fold of control, which was set to 1. Values represent the mean ± SD of two independent experiments. (**B**) Regulation of IL-6 quantified by ELISA. *p < 0.05 was considered statistically significant by ANOVA followed by Tukey's multiple comparisons test.

In recent works, primary hPDLF respond to compressive force with an upregulation of pro-inflammatory markers^22–24^. However, this is not well-known for cementoblast cells yet. We therefore analyzed the expression of the pro-inflammatory genes *Il-6, Il-1a, Cox2, Vegfa* and *Mmp9*. We found a significant increase of *Il-6* and *Vegfa* during the course under compressive force of our experiments. Regarding *Il-6*, XN had no effect on basal gene expression, but under loading compression it was significantly upregulated. Xanthohumol decreased compression related upregulation of *Il-6*, but the expression was still higher compared to control. In contrast, XN had no effect on *Il-1a* and *Cox2,* neither on basal level nor in case of compressive force. However, both genes were significantly upregulated in loaded probes. Additionally, *Vegfa* and *Mmp9* showed minor regulations within the experimental conditions (Fig. 3A).

### Xanthohumol re-established the levels of IL-6 protein expression from compressively stimulated cementoblasts to basal conditions

To prove the upregulation of *Il-6,* observed in gene expression, we measured protein levels of IL-6 by ELISA. After addition of compressive strain, IL-6 was significantly upregulated. Furthermore, we confirmed that IL-6 expression was reduced by addition of XN under compressive force. XN decreased the basal IL-6 expression without reaching a significant level. This effect was again significantly decreased by XN without reaching the control level (Fig. 3B).

### Xanthohumol re-established the phosphorylation of ERK and AKT initiated by compressive stimulation

**Figure 4:**
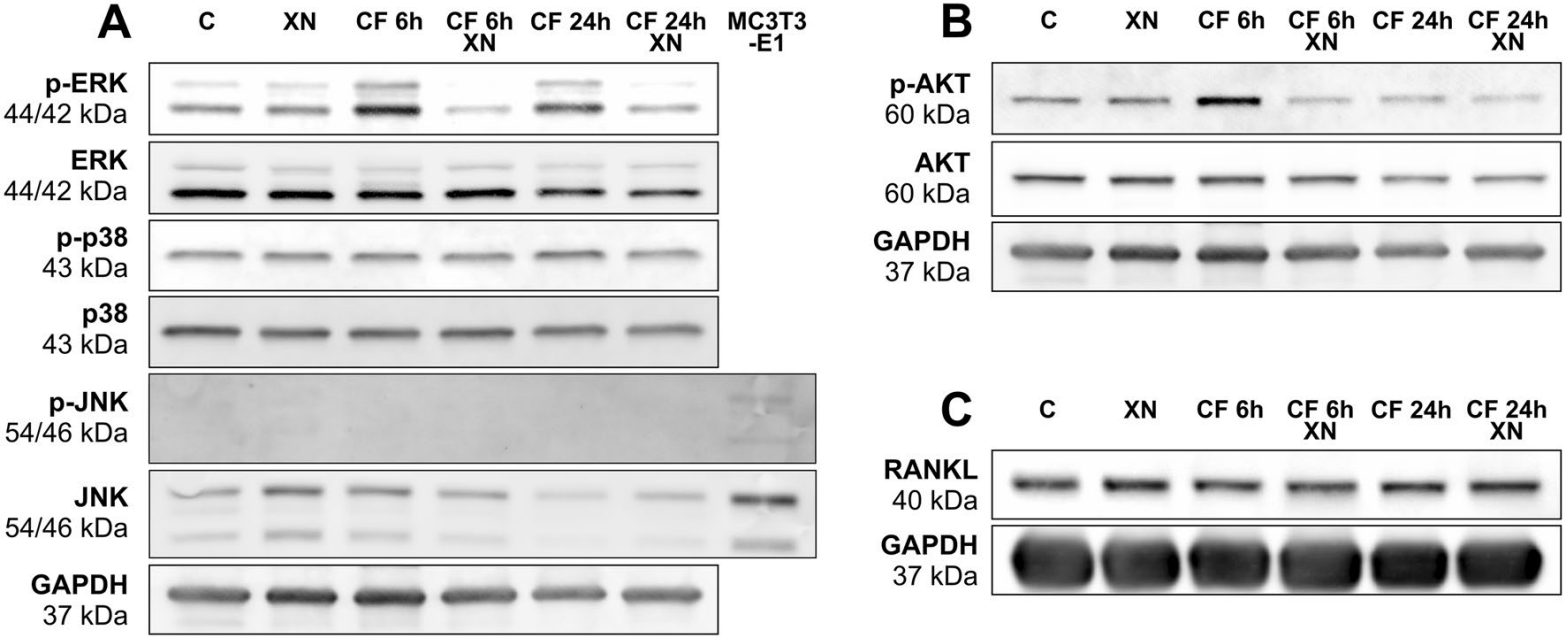
Effects of xanthohumol (XN) on expression and phosphorylation of MAP-kinases ERK, JNK and p38 (**A**), AKT (**B**) and RANKL (**C**) in mechanically stimulated OC/CM cells. OC/CM were pretreated with XN at final concentration of 0.4 µM for 24 h, respectively, followed by static compressive stimulation for 24 h. Representative blots of three independent experiments are presented. GAPDH was used as loading control. Phosphorylated variants are labeled as “p- “. MC3T3-E1 cells, activated by UV radiation for 30 min served as positive control for JNK and p-JNK. CF = stimulation with static compressive force (see Supplementary Fig. S1 online for uncropped blots).

The cytokine expression under inflammatory process in cementoblasts hasn’t been explained yet. To this purpose, we investigated some possible pathways involved in cytokine expression, such as MAP-kinases (ERK, p38 and JNK) and protein kinase B (AKT) and their activated/phosphorylated form, by western blot. Phospho-ERK and phospho-AKT were clearly upregulated in probes with mechanical strain both after 6 h and after 24 h. Addition of XN re-established the upregulation up to a level compared to basal conditions, similar to control. Furthermore, XN tended to reduce phosphorylation of ERK and AKT in unstimulated probes without having any effect on the non-phosphorylated expression (Fig. 4A,B). MAP-Kinase p38 was expressed in OC/CM cells but no changes were observed in phosphorylation status by mechanical compression or after XN application. However, only JNK expression could be observed and the phosphorylated form of JNK was not detectable in OC/CM cells neither with mechanical stimulation nor after addition of XN (Fig. 4A). Receptor Activator of NF-κB Ligand (RANKL) was detectable and slightly upregulated by mechanical stimulation without being affected by XN (Fig. 4C).

## Discussion

Until now, multiple effects of XN, such as anti-inflammatority^25,26^, antibacteriality^27^, anti-angiogenity^26,28^ and anti-proliferativity^28^ have been described. Concerning the periodontal microenvironment, anti-inflammatory mechanisms are of highest interest, since compression related remodeling processes in this area are associated with a local sterile inflammation^29^. The effect of XN on bone resorption may also be profitable in other inflammation types in periodontal tissues without compressive stimuli, like periodontitis. In this work, we investigated the effects of Xanthohumol on immortalized cementoblasts during compressive force application, hypothesizing that the anti-inflammatory properties may modulate the inflammatory signaling processes. The drug concentrations corresponded to typical concentrations in standard beer (up to 0.4 µM)^30^ and XN enriched sorts of beer (8.5 µM)^31^. In addition, these concentrations were in a comparable range with total human XN blood plasma concentrations (free and conjugated) after oral XN administration^21^. The proportion of conjugated XN relative to free XN in human plasma is close to 100% for conjugated XN^21^. In many cases, conjugated agents, in contrast to free ones, are not relevant for therapeutic effects. Nevertheless, in case of XN, biological effects seem to correlate with total (free plus conjugated) XN levels, not only with the levels of free XN^21,32,33^. Furthermore, XN has been shown to have a high bioavailability and a low toxicity profile^34,35^. Orally administered Xanthohumol does not affect major organ functions *in vivo*^36^.

The in vitro experiments in this study showed dose dependent effects of XN on cytotoxicity and cell viability. Low doses increased cell viability while higher doses provoked cytotoxic effects. Based on this, a XN concentration of 0.4 µM has been used for further experiments with compressive stimulation. Higher concentrations were not investigated in further experiments on mRNA and protein level due to high significance and better clinical plausibility of 0.4 µM. The level of exerted force was similar to previous experiments with fibroblasts of the periodontal ligament^37,38^ and cementoblasts^39,40^. In these experiments, an initial lag phase of 24 h after force application followed by continuing proliferation has been reported^37^. We were able to confirm decreased cell proliferation in compressed cementoblasts by cell cytometry. XN had supporting effects on cell proliferation and survival, comparable to our findings regarding cell viability.

Comparing compressively stimulated cementoblasts with their control, we were able to confirm the results from previous publication indicating that *Cox-2* gene expression is upregulated by loading compression^41^. Our findings regarding compression related *Il-6* upregulation correlate with previous publications with periodontal ligament fibroblasts^42^. IL-6 is an important marker of periodontal remodeling, being involved in osteoclast differentiation and activation^17^. This regulation indicates that cementoblasts are involved in regulatory mechanisms after compressive strain. As hypothesized, XN is able to decrease this compression dependent IL-6 upregulation on mRNA and protein level. Regarding anti-angiogenic properties of XN, we further investigated the regulation of *Vegfa* that is upregulated in PDL cells^13^ and not explored in compressively stimulated cementoblasts yet. We did not observe any relevant regulations neither of compressive force nor of XN on *Vegfa* mRNA expression. In contrast, no effects on other proinflammatory markers´ gene expression, such as *Il-1a, Vegfa, Mmp9* and *Cox2,* were detectable.

Despite the exact intracellular target of XN and its mechanism remains unclear, previous studies with various cancer cell lines have demonstrated that XN interacts with the three MAP-Kinase subfamilies: ERK^43–45^, JNK^45^ and p38^46^. XN effects on protein kinase B/AKT are known too^47–49^. The well-known relevance of these kinases for cell proliferation, cell survival, cell stress and inflammatory responses is important for getting a first insight in XN related changes in cementoblasts. Our data provide evidence that XN may influence cell proliferation and cell survival, as seen in cell cytometry experiments (Fig. 2) and gene and protein expression of IL-6 (Fig. 3). As we reported before, phospho-ERK and phospho-AKT are upregulated by compressive stimulation^40^ and—corresponding to publications about XN in cancer cell lines—both phosphorylated forms were decreased by XN. Activated ERK is able to translocate into the nucleus and modulates genes for the response to stimulation^50,51^. Taking this together, we hypothesize a correlation of ERK activation and IL-6 expression caused by mechanical stimulation in cementoblasts. Regulations of phospho-ERK following mechanical strain are also known in PDLF^52^. In contrast to that, JNK, p38 and their phosphorylated forms were not altered neither by mechanical stimulation nor by XN. This suggests first indications that these kinases are not involved in the cellular reaction to compressive stimuli in cementoblasts. Finally, we investigated possible effects of XN on RANKL protein expression. RANKL is a key molecule for bone regeneration and remodeling by affecting the differentiation of osteoclasts^53^. As we showed, static compressive stimulation had just a slight effect and XN had no impact on RANKL. This expression, nearly independent of mechanical stimulation, contrasts with previous studies with human PDLF^54^ and cementoblasts^41^, where a clear upregulation was observed.

In summary, we were able to demonstrate selective anti-inflammatory effects of Xanthohumol in murine cementoblasts. This may be an interesting option for the modulation of inflammatory responses within the PDL in in vivo studie*s* for orthodontic therapy and treatment of periodontitis.

## Material and methods

### Reagents and antibodies

Xanthohumol (#2828.1, purity > 99%, Carl Roth, Germany) was solved in Dimethylsulfoxid (DMSO) (Carl Roth, Germany) and stored at − 20 °C in 10 mM aliquots. Each aliquot was used a single time to avoid freeze/thaw degradation cycles. The primary antibodies Phospho-Akt (Ser473)(D9E) dilution 1:2000 (#4060); Phospho-p44/42 MAPK (Erk1/2) (Thr202/Tyr204) (D13.14.4E) dilution 1:2000 (#4370); Phospho-p38 (Thr180/Tyr182) dilution 1:2000 (#9216S); Phospho JNK (Thr183/Tyr185) dilution 1:2000 (#9255S); AKT (40D4) dilution 1:2000 (#2920); p44/42 MAPK (Erk1/2) (3A7) dilution 1:1000 (#9107); p38 MAPK (D13E1) dilution 1:1000 (#8690S); JNK dilution 1:1000 (#9252S); GAPDH (14C10) dilution 1:1000 (#2118S) were purchased from CellSignaling, USA. Secondary antibodies StarBrightBlue700 (12004158) and StarBrightBlue 520 (12005869) were purchased from BioRad,USA. For Western blotting, RIPA-buffer (ThermoFisherScientific,USA) complemented with cOmplete Tablets Mini and PhosStop (Roche,Swizerland). Carboxyfluoreszein‐succinimidyl ester (CFSE) was purchased from Invitrogen (Carlsbad, CA, USA), while Propidium ionide solution (PI) was provided by Miltenyi (Miltenyi Biotec, Germany).

### Cell culture

Immortalized murine osteocalcin expressing cementoblasts (OC/CM), kindly provided by Prof. Somerman^55,56^, were cultured in DMEM low glucose (1 g/L)(Gibco, USA), 10% FCS (Gibco, USA), 100 units/mL of penicillin and 100 μg/mL of streptomycin (Gibco, USA) in cell culture plates at 37 °C and 5% CO2 in a humidified atmosphere. Cells were trypsinized, centrifuged at 350 g, quantified using a Neubauer Counting Chamber and 80,000 cells were plated in each 6-well for quantitative realtime-RT-PCR analysis and western blotting. When reaching a confluence of 90%, cells were mechanically compressed with glass cylinders, which exert a static force (2 g/cm^2^) for 6 h and 24 h on the cell monolayer. This compression method has already been established for cementoblasts and described in previous publications^39,40,57,58^. All experiments were performed with DMEM, 1% FCS.

### MTS assay

For MTS-assay 5000 cells/well were seeded in 96 well plates in 50 µl 1% FCS medium. XN concentrations from 0.2 µM up to 8 µM were added after 24 h of acclimatization by adding 50 µl of medium with double XN concentration than needed to obtain correct concentrations in the wells. Then, after 6 h, 12 h, 24 h and 48 h, 20 µl of CellTiter 96® AQueous One Solution Cell Proliferation Assay that contains 3-(4,5-dimethylthiazol-2-yl)-5-(3-carboxymethoxyphenyl)-2-(4-sulfophenyl)-2H-tetrazolium (MTS) (Promega, USA) were subjoined. After 2 h of incubation, absorption was measured according to manufacturers’ protocol using an ELISA plate reader (Tecan, Switzerland). Cell viability was calculated relative to control.

### Isolation and purification of RNA

For RNA-isolation cells in each well were first washed with 2 mL phosphate-buffered saline (Gibco) and then harvested with 0.5 mL TRIzol™ Reagent (Thermo Fisher Scientific, USA), two wells were pooled. This leads to biological triplicates for each condition. After isolation, according to the man-ufacturer’s instructions, the RNA yield of each sample was verified photometrically at 280 nm and 260 nm (Nanodrop One™, Thermo Fisher Scientific, USA). Afterwards RNA purification was per-formed with RNeasy Mini Kit (Qiagen, Germany) following the producers’ protocol including an on-column DNA digestion (RNase-Free DNase, Qiagen, Germany). In order to control the success of the purification and to ensure a uniform cDNA synthesis, each sample was measured again (Nanodrop One™).

### RT-qPCR

The RNA was transcribed into cDNA (SuperScript III RT, Thermo Fisher Scientific, USA). Basing upon the measurement after RNA purification, the final concentration was 25 ng/μl. All steps from RNA isolation to cDNA synthesis were performed in parallel for all samples of each experiment in order to avoid experimental variations. RT-qPCR was performed in technical duplicates using 2.5 ng/μl cDNA in each reaction and a primer concentration of 0.5 μM. The qTower3 (Analytik Jena, Germany), High Green Mastermix (Thermo Fisher Scientific, USA), qPCR-Soft 3 (Analytik Jena, Germany) and self-designed intron spanning primers (Eurofins, Luxembourg) were used. Primers were designed by using Primer-BLAST (NCBI, USA) followed by a PCR-Check (Eurofins Oligo Analyse Tool, Luxembourg) to ensure in silico qPCR specificity. Our criteria were length ca. 20 bp, annealing temperature 60 °C, max product length 200 bp, intron spanning, covering possible transcript variants (Table 1). The RT-qPCR protocol included an initial step of 50 °C for 2 min, 95 °C for 10 min followed by 40 cycles of 95 °C/15 s, 60 °C/30 s and 72 °C/30 s. A step of 95 °C for 15 s forms the transition to melting curve analysis (60–95 °C). All RT-qPCR data were normalized by delta-delta Ct method to the reference gene *Rpl22,* that we have validated in a previous publication^39^ and to the unstimulated control.

**Table 1:** RT-qPCR gene, primer and target/amplicon information for the reference gene *Rpl22* and investigated target genes.

Gene symbol	Gene name (mus musculus)	Gene function	Accession Number (NCBI Gene Bank)	Chromosoma location (length)	5′-forward primer-3′ (length/Tm/%GC)	5′ reverse primer-3′ (length/Tm/%GC)	Primer location	Amplicon length	Amplicon location (bp of start/stop)	Intron-flanking (length)	Variants targeted (transcript/splice
*Rpl22*	Ribosomal protein L22	Translation of mRNA in protein	NM_001277113.1	4; 4 E2 (2153 bp)	AAGTTCACCCTG ACTGCAC (20 bp/60.18 °C/55%)	AGGTTGCCAGCTT TCCCATT (20 bp/60.18 °C/50%)	Exon 2/3	110bp	166/275	Yes	Yes
*Il-6*	Interleukin 6	Important role in bone metabolism; osteoclastogenesis	NM_031168.2	5 B1; 5 15.7 cM (1083 bp)	ACTTCACAAGTCGG AGGCTTA (21 bp/59.03 °C/47.62%)	TTTTCTGCAAGTGCA TCATCGT (22 bp/59.45 °C/40.91%)	Exon 2/3	116 bp	220/335	Yes	Yes
*Il-1a*	Interleukin 1a	Important role in bone metabolism; osteoclastogenesis	NM_010554	2 F1; 2 62.9 cM (1974 bp)	GCCATTGACCATT CTCTCTGA (22 bp/59.57 °C/50%)	TGATACTGTCACC CGGCTCT (20 bp/60.32 °C/55%)	Exon 3/4	156 bp	130/285	Yes	Yes
*Vegfa*	Vascular endothelial growth factor A	Induces proliferation and migration of vascular endothelial cells	NM_001025250	17 C; 17 22.79 cM; ( 3547 bp )	TCTCCCAGATCGT GACAGT (20 bp/59,96 °C/55%)	AAGGAATGTGTGT GGGGAC 20 bp/59,89 °C/55%)	Exon 8	98 bp	3022/3119	No	Yes
*Mmp9*	Matrix metallo- peptidase 9	Breakdown of extracellular matrix, reproduction, and tissue remodeling	NM_013599.4	2 85.27 cM; (3189 bp)	CCCTGGAACTCC ACGACAT (20 bp/59.86 °C/55%)	TGGTTCACCTCAT GGTCCAC (20 bp/59.6 °C/55%)	Exon 12–13	119 bp	2064/2182	Yes	Yes
*Ptgs2 (Cox2)*	Prostaglandin-endoperoxide synthase 2	Involved in prostaglandin synthesis	NM_011198.4	MT (non nuclear) (4460 bp)	TGAGTACCGCAA CGCTTCT (20 bp/59.97 °C/50%)	GCAGGGTACAGTTC ATGACA (21 bp/60 °C/52.38%)	Exon 9/10	126 bp	1543/1668	Yes	–

Tm, melting temperature of primer/specific qPCR product (amplicon); %GC, guanine/cytosine content; bp, base pairs; MT, mitochondrial.

### Combined proliferation and cell death assay

OC/CM cells were stained with 2.5 µM CellTrace™ CFSE Cell Proliferation Kit (Thermo Fisher Scientific, USA) according to the instructions of the supplier. 200,000 cells were seeded in 6-well cell culture dishes in complete medium. After 8 h incubation, cells were treated with 0.4 μM of xanthohumol for 48 h, combined with and without compression in the last 24 h. Thereafter, floating and attached cells were collected, resuspended in 400 μl PBS with 2% FCS and analyzed by flow cytometry, FACS Canto (BD Biosciences, USA). The number of viable cells was assessed for 60 s in same volume and at constant speed. Proliferation was traced by CFSE staining and normalized to the control, while cell death was determined by staining with PI.

### Westernblot

For extraction of cellular proteins, OC/CM were lysed on ice with Pierce RIPA buffer with added cOmplete Tablets Mini and PhosStop. Subsequently, lysate was centrifuged (10 min, 20 000 g, 4 °C). The protein concentration of cell lysates was analyzed by Bradford assay (Bio-Rad, USA). Proteins (15 µg) were loaded onto 12% SDS–polyacrylamide gels (#1610185, Bio-Rad, USA) and transferred onto PVDF membranes 0.2 µm (#1704156, Bio-Rad, USA). After being blocked in 5% BSA in TBS-T (TRIS-buffered saline and 0.1% Tween-20) for one hour at RT, membranes were incubated at 4 °C overnight with primary antibodies. The immunoreactive bands were detected by using fluorescent secondary antibodies with the ChemiDoc MP imaging system (Bio-Rad, USA).

#### ELISA

To analyze the translational level, a commercially available enzyme linked immunosorbent assay (ELISA) kit for IL-6 (# 88-7064-22, Thermo Fischer Scientific, USA) was used following manufacturers’ instructions with fresh cell culture supernatant.

### Statistical analysis

First, data were checked for normal distribution by Shapiro–Wilk test and for homogeneity of variance by Brown-Forsythe test followed by an ANOVA analysis of variance with Tukey’s post hoc test (Figs. 2, 3, 4) or Welch’s ANOVA with Dunnett's T3 multiple comparisons test due to unequal variances (Fig. 1). (Prism version 9.0.0; GraphPad Software), where *p* < 0.05 was considered statistically significant. Graphs show mean values ± standard deviation (SD).

## Supplementary Information

Supplementary Information.

## Data Availability

The data that support the findings of this study are available from the corresponding author R.B.C. upon reasonable request.
